# Exploring the variation of emotional intelligence in health sciences students: A mixed method study across pre-clinical and clinical years

**DOI:** 10.12669/pjms.41.11.10255

**Published:** 2025-11

**Authors:** Imran Khalid, Kinza Aslam, Saqib Siddiq Choudhry, Rehan Ahmed Khan

**Affiliations:** 1 Imran Khalid, M.Phil. Orthoptics (KEMU), MME (UOL), Services Institute of Medical Sciences, Lahore, Pakistan; 2 Kinza Aslam, MME (UOL) Associate Professor, Medical Education, The University of Lahore, Lahore, Pakistan; 3 Saqib Siddiq Choudary, FCPS Professor of Ophthalmology, Services Institute of Medical Sciences, Lahore, Pakistan; 4 Rehan Ahmed Khan, FRCS, PHD-HPE Professor of Surgery, Riphah International University, Islamabad, Pakistan

**Keywords:** Emotional intelligence, Soft skills, Health sciences students, Pre-clinical and clinical years, MBBS, BDS, Physiotherapy, Optometry

## Abstract

**Objective:**

To assess the variation in levels of emotional intelligence of students in pre-clinical and clinical years of health sciences and to explore what factors were associated with differing levels of EI.

**Methodology:**

This mixed-method study was done at the University of Lahore from March to August 2023. Quantitative data was collected using the Trait Emotional Intelligence Questionnaire-Short Form (TEIQue-SF) from 248 students, 62 participants were recruited from each discipline of health sciences students. Three focus group discussions and four one-on-one interviews were performed. Thematic analysis of the qualitative data was done based on Daniel’s Goleman model of EI’s theoretical framework.

**Results:**

One-third (32.30%) of health sciences students had low EI levels. Physiotherapy clinical years had a higher mean EI score (141.13 ± 13.42 SD). In MBBS clinical years, EI decreased (p> 0.02), while for BDS, physiotherapy, and optometry, it remained non-significant (p> 0.05). However, proportion of students with moderate EI declined in MBBS (from 67.7% to 41.9%) and optometry (from 83.9% to 67.7%) but increased in physiotherapy (from 64.5% to 77.4%) and BDS (from 58.1% to 61.3%). Five key factors were identified influencing EI levels: self-awareness, social awareness, teacher role modelling, self-management, and communication skills.

**Conclusion:**

Students’ EI varied across and within disciplines, depending on self-awareness, the working environment, and the responses of their teachers and patients. Soft skills should be explicitly incorporated into the curriculum and reinforced through positive role modelling by teachers.

## INTRODUCTION

Emotional intelligence (EI), as defined by Daniel Goleman, is “the capacity for recognizing our own feelings and those of others, for motivating ourselves, for managing emotions well in ourselves and in our relationships”.^1^ Research has shown that EI positively impacts students’ performance in both academics and health care delivery in clinical settings.^2^ This emphasizes the importance of EI in the development of effective healthcare professionals who can manage not only their own emotions but also understand and respond appropriately to the emotions of their patients.

Studies indicate varied levels of EI among health sciences students reflecting both the complexity of EI and the diverse educational environments. For instance, EI was found to be at good level in 21.6% of MBBS students and 19.71% of physiotherapy students suggesting that a significant proportion of students in these fields may benefit from additional support in developing their EI skills.^2^-^3^ Another study done on MBBS and BDS students revealed a high level of EI in only 54.9% of the participants.^4^ A systematic review spanning from 1980 to 2018 indicated that EI decreased over the years of study in MBBS students,^5^ whereas another study found that EI remained consistent over the years of study.^6^ In optometry students, no significant difference was observed in EI between pre-clinical and clinical years.^7^ An interdisciplinary study showed higher levels of EI in MBBS students compared to other health sciences students^8^, while another study found no difference in EI levels among students from various health sciences students disciplines.^9^

The objective of this study was formulated based on controversies in the current literature whether the EI decreased or remains constant over the years of study and the non-homogenous sample size distribution were employed.^2^-^9^ Furthermore, there is a paucity of qualitative research exploring students’ understanding, beliefs, and perceptions of EI of across different health sciences disciplines.^5^-^9^ This study aimed to fill that gap by employing mixed methods study design to investigate the variation and associated factors in differing the EI between pre-clinical and clinical years students of MBBS, BDS, optometry, and physiotherapy disciplines.

## METHODOLOGY

This mixed-methods study included undergraduate students from four health sciences disciplines: MBBS, BDS, physiotherapy, and optometry. The sample size of 248 students was calculated using the OpenEpi calculator with a 95% confidence interval. Sixty-two students from each discipline were selected and categorized equally into pre-clinical (N = 31) and clinical years (N = 31). Every third student from the second year (pre-clinical) and final year (clinical) was selected through systematic random sampling. First year students were excluded because they had limited clinical exposure^10^ whereas third year students were excluded due to significant decline in motivation during this stage.^11^ This study was approved by the ethical review board of the University of Lahore (Ref: ERC/08/23/01; Dated: February 3, 2023) and adhered to the Declaration of Helsinki.

For the quantitative phase, data was collected using a validate questionnaire Trait Emotional Intelligence Short Questionnaire (TEIQue-SF) having Cronbach’s alpha of 0.88 and consisting of 30 items rated on a 1-7 Likert scale from entirely disagree to agree, with a cumulative score of 210.^8^ Low EI was defined as a score <60% (<126), moderate EI as 60%-80% (126-167), and high EI as >80% (168-210).^10^

The quantitative data was analyzed using SPSS-24. Within the MBBS discipline, the Mann-Whitney U test was applied to analyze abnormally distributed data. The independent sample t-test was used for normally distributed data for BDS, optometry, and physiotherapy. A Chi-square test was applied to compare the levels of EI against ordinal variables (low, moderate, and high).

Purposive sampling was employed for the qualitative phase which included three focus group discussions (FGDs) with dentistry, optometry, and physiotherapy students (coded as FGD1-FGD3) and four one-on-one interviews with MBBS students (coded as Participant A-D).

An interview guide validated by medical education experts were used in all sessions. To ensure reflexivity and participants comfort, interviews allowed withdrawal from any question, could be conducted face-to-face or online and permitted responses in any language. All interviews were manually transcribed to ensure transferability, and an audit trail of coding decisions and data analysis procedures was maintained to ensure the confirmability of the research process. An initial coding framework was developed inductively and then refined through discussion until consensus was reached. Inter-coder reliability was established by cross-checking and resolving discrepancies collaboratively. Data saturation was determined when no new codes or themes emerged after three consecutive interviews.

Daniel Goleman’s framework was used as a theoretical lens to structure and interpret the data. While the initial codes were inductively derived, the emergent themes were subsequently mapped onto Goleman’s dimensions of emotional intelligence (self-awareness, social-awareness, self-management and social management). This ensured that the analysis was both grounded in the data and theoretically informed.

The semi-structured interview was based on the following questions: What are the emotions you experienced in your academic and clinical work? What are your strengths and weaknesses in academic and clinical work? What kind of behavior do you observe from your teachers toward patients? How do communication skills relate to emotional management? How is the role modeling of teachers in the early clinical years influencing emotional intelligence?

## RESULTS

Out of 248, 32.30% (80) of Health Sciences students had low levels of EI. Students had a moderate EI score of 65.30%. A higher mean score of EI (141.13 ± 13.42 SD) was found in the clinical years of physiotherapy. The EI score of MBBS students significantly decreased (p<0.024) in clinical years (123±18 SD) compared to pre-clinical years (134±15 SD). While in the other three disciplines (BDS, physiotherapy, and optometry), the difference in EI was not statistically significant (p>0.05), (Table-I). However, the moderate EI of clinical years of MBBS students decreased from 67.7% (21) to 41.9% (13) and optometry from 83.9% (26) to 67.7% (21). The EI of physiotherapy students in the clinical year increased from 64.5% (20) to 77.4% (24), and BDS from 58.1% (18) to 61.3% (19) (**Table-II).** The qualitative phase provided insights that contextualize five themes, sixteen subthemes and forty-seven codes were emerged and the following factors were associated with different levels of EI in students.

**Table-I:** Comparing the variation of EI Score of pre-clinical versus clinical years.

		N	Mean / median	Std. Dev / IQR	Std. Error Mean	P value
***MBBS***	Pre-clinical years	31	134	15	2.238	0.024
	Clinical years	31	123	18	2.99	
***BDS***	Pre-clinical years	31	129.48	15.063	2.797	0.701
	Clinical years	31	130.97	15.171	2.641	
***Physiotherapy***	Pre-clinical years	31	134.35	20.972	3.767	0.135
	Clinical years	31	141.13	13.421	2.41	
***Optometry***	Pre-clinical years	31	139.06	14.294	2.567	0.293
	Clinical years	31	134.87	16.721	3.003	

**Table-II:** Comparing the levels of EI in pre-clinical year and clinical years.

Disciplines		Years of Study	Levels of emotional intelligence			Total	P-value
			Low	Moderate	High		
***MBBS***	Pre-clinical years		10	21	0	31	0.096
			32.3%	67.7%	0.0%	100.0%
	Clinical years		17	13	1	31
			54.8%	41.9%	3.2%	100.0%
***BDS***	Pre-clinical years		13	18		31	0.796
			41.9%	58.1%		100.0%
	Clinical years		12	19		31
			38.7%	61.3%		100.0%
***Physiotherapy***	Pre-clinical years		9	20	2	31	0.471
			29.0%	64.5%	6.5%	100.0%
	Clinical years		5	24	2	31
			16.1%	77.4%	6.5%	100.0%
***Optometry***	Pre-clinical years		5	26	0	31	0.263
			16.1%	83.9%	0.0%	100.0%
	Clinical years		9	21	1	31
			29.0%	67.7%	3.2%	100.0%
	Total		80 (32.2%)	162(65.32%)	6 (2.41%)	248 (100%)	

### Self-awareness:

Students described difficulties in recognizing and managing their own emotional responses during patient care, which may partly explain the drop in EI among clinical-year MBBS students. One participant said, *“When I see a patient with a very bad condition… I messed up whole”* (Participant A), while another added, *“I was emotionally drained… it’s very difficult for me to continue to smile and show welcoming behavior”* (FGD1, P3). These reflections indicate that clinical encounters challenge students’ emotional resilience, aligning with the lower EI scores observed in MBBS.

### Social awareness:

Empathy and organizational climate were noted as significant factors. For example, *“A patient feels autonomy as he wants some good doctor, a good doctor must be anxiety-free”* (FGD1, P3). Another participant highlighted how external factors shaped their emotions: *“Co-operation of colleagues, comfortable climate and weather condition of my classroom and my skill lab affect my mood a lot”* (FGD1, P5). Such accounts suggest that institutional and interpersonal contexts strongly influence EI levels.

### Self-management:

Stress management was viewed as essential, yet often difficult in clinical settings. *“We have to give priority to patients irrespective of whatever emotions we have”* (FGD3, P1). This explains why some students were able to maintain or even improve EI (as in physiotherapy), while others struggled.

### Role modeling:

Students emphasized the dual effect of positive and negative role models. Supportive teachers inspired growth (*“we idealize our teachers and want to become just like them”* - FGD1, P4), while negative experiences undermined confidence (*“This kind of teacher’s behavior negatively impacted me… and I started to hesitate to ask a question”* - FGD2, P6). This divergence may help explain why physiotherapy students (reporting supportive mentors) improved in EI, whereas MBBS students (reporting discouraging experiences) declined.

### Communication

Compassionate communication from teachers was seen as a driver of EI: *“If a teacher compassionately communicates… this will positively affect the learners”* (FGD3, P1). In contrast, unsupportive communication discouraged engagement and learning.

## DISCUSSION

Some studies have found that emotional intelligence decreases during clinical years, while others suggest that EI remains stable throughout the course of study.^5^-^9^ However, many of these studies have employed unequal and convenience sampling methods.^4^-^12^ In contrast, the current study utilizes, equal sample size distribution and systematic random sampling. This approach enhances the likelihood of equal representation across different health sciences disciplines and facilitates broader generalization of the results.

One third of health sciences students were found to have a low level of EI. In contrast, an Indian study reported half of the students were in the low category. This difference in high proportions might be because their study had only two categories (below average and above average), while our study classified EI into low, moderate, and high.^12^

This study confirmed that the EI statistically significant P-value was <0.05 in clinical years of MBBS compared to pre-clinical. These findings are consistent with the two independent studies, which claimed that the EI of MBBS students decreased in clinical years.^5^,^13^ This decrease in EI could possibly be due to variations in clinical presentations of signs and symptoms in the real setting. As one participant said: *“Emotions are inevitable, as the patient is in distress situation and I get really annoyed in critical conditions”* (FGD1, P1). Other potential explanations for the declined in EI, as highlighted by Ewaiwe, et al. include change in sign and symptoms between book description and actual clinical presentations, and competition to work more to get paid residence.^13^ The P-value >0.05 was non-significant in the optometry, physiotherapy, and BDS disciplines. However, a significant percentage of EI decreased in the clinical years of optometry and, conversely, increased in physiotherapy and BDS students. That’s why psychologists do not rely solely on P-values as a final tool.^14^

In our study, a new subtheme of the coexistence of strength and weakness emerged, which means the strength of a person at one time becomes the weakness on some other occasion. One of the study participants said that *“In clinical work, we have to tolerate the patients but sometimes this tolerance becomes our weakness as well”* (FGD3, P3). The study done by Chandran only highlighted strengths and weaknesses in the domain of self-awareness. Through self-regulation, students can tailor their emotions by recognizing their strengths and weaknesses.^15^ Our study highlighted that student adapted well to various situations, as one of the participants said *“We have to respect the teacher, even if we are feeling angry”* (FGD3, P1). Lal and Diyal found that EI positively correlated with social adaptation.^16^

Students cannot regulate their positive emotions after a specific time as their emotions drain. A clinical supervisor remembers that half of the students’ burnout occurs after 40 work hours per week.^17^ Some students feel uncomfortable working in large groups and communicating with peers, teachers, and patients. *“My weakness is that I cannot work in the large group”***(Participant A).** This weakness can be addressed by fostering teamwork and assigning group tasks. Additionally, teamwork helps manage conflicts and improves history-taking, empathetic listening, and communication skills.^18^

About 50% of students identified at least one of their medical teachers as a negative role model, as one of the participants said *“Harsh behavior of my teacher set me back emotionally when I started to work on a dental chair”* (FGD1, P3). This negative role modelling led many students to lose interest in their studies, confidence, communication skills, and self-esteem.^19^ Therefore, both teachers and students must reflect on and reevaluate their essential soft skills to enhance components of emotional intelligence, such as self-awareness, empathy, communication skills, teamwork, adaptability, and tolerance, to better cope with academic and clinical challenges.^20^ Hence, this study has depolarized the existing dichotomy that EI decreased or remained stable over the years of study.^5^,^8^,^13^

### Limitations

The study’s limitation is that it was conducted at a private university and did not compare the EI of public sector students. Two independent studies showed a great deal of controversy; one claimed the high EI of private sector students, while the other favored the public sector.^4^,^12^ So, to enhance the credibility and generalizability of these results, it is imperative to conduct further research in a multicenter study setting involving both public and private institutions.

## CONCLUSION

One-third of health sciences students had low emotional intelligence. Students’ EI varied across and within disciplines, depending on self-awareness, the working environment, and the responses of their teachers and patients. Soft skills such as empathy, teamwork, communication, and self-management should be explicitly incorporated into the curriculum and reinforced through positive role modeling by teachers.

### Authors’ Contributions:

**IK and RAK:** Conceptualized, designed, acquisition, analysis and interpretation of data.

**IK, KA and RAK:** Literature search, critical analysis, Final approval of manuscript.

**SSC:** Manuscript drafting & revision for intellectual content.

**RAK:** Was the supervisor of this research work.

All authors have read and approved the final version of the manuscript
